# Autopsy findings of acute erythroid leukemia

**DOI:** 10.4322/acr.2023.429

**Published:** 2023-05-08

**Authors:** Mayur Parkhi, Nabhajit Mallik, Deepesh Lad, Man Updesh Singh Sachdeva, Amanjit Bal, Pankaj Malhotra, Suvradeep Mitra

**Affiliations:** 1 Postgraduate Institute of Medical Education and Research, Department of Histopathology, Chandigarh, India; 2 Postgraduate Institute of Medical Education and Research, Department Hematology, Chandigarh, India; 3 Postgraduate Institute of Medical Education and Research, Department of Clinical Hematology and Medical Oncology, Chandigarh, India

**Keywords:** Pancytopenia, Leukemia, Erythroblastic, Acute, Diagnosis, Differential, Tumor Suppressor Protein p53, Autopsy

## Abstract

Acute erythroid leukemia (AEL) is an exceedingly uncommon but distinct hematological malignancy that shows neoplastic proliferation of erythroid precursors with maturation arrest and no significant myeloblasts. We describe an autopsy case of this rare entity in a 62-year-old man with co-morbidities. He underwent a bone marrow (BM) examination for pancytopenia during the first outpatient department visit, which revealed an increased number of erythroid precursors with dysmegakaryopoiesis suggesting the possibility of Myelodysplastic syndromes (MDS). Thereafter, his cytopenia got worsened, warranting blood and platelet transfusions. Four weeks later on the second BM examination, AEL was diagnosed based on morphology and immunophenotyping. Targeted resequencing for myeloid mutations revealed TP53 and DNMT3A mutations. He was initially managed along febrile neutropenia with the stepwise escalation of antibiotics. He developed hypoxia attributed to anemic heart failure. Subsequently, he had hypotension and respiratory fatigue pre-terminally and succumbed to his Illness. A complete autopsy showed infiltration of various organs by AEL and leukostasis. Besides, there was extramedullary hematopoiesis, arterionephrosclerosis, diabetic nephropathy (ISN-RPS class II), mixed dust pneumoconiosis, and pulmonary arteriopathy. The histomorphology of AEL was challenging, and the differential diagnoses were many. Thus, this case highlights the autopsy pathology of AEL, an uncommon entity with a strict definition, and its relevant differentials.

## INTRODUCTION

Acute erythroid leukemia (AEL) is a highly uncommon and distinct entity representing less than 1% of all acute myeloid leukemia (AML) cases.^1,2^ It is characterized as a neoplastic proliferation of erythroid cells with features of maturation arrest, increased erythroblasts, and a high prevalence of biallelic *TP53* alterations.^3^ It was previously termed pure erythroid leukemia (PEL), which is acceptable and interchangeable in the latest 5th edition of the World Health Classification of the Hematolymphoid Tumours.^3^ The disease is mainly confined to the peripheral blood and bone marrow. The essential diagnostic criteria include erythroid predominance, usually ≥80% of bone marrow elements, of which ≥30% are erythroblasts.^3^ It may arise de novo or following myelodysplastic neoplasms (MDS) or MDS/MPN (myeloproliferative neoplasms), and both conditions share distinctive morphologic features, with prominent proerythroblast proliferation.^4,5^ AEL has a rapid and aggressive clinical course with no standard treatment guidelines and has a dismal prognosis (median survival of 1.8 months).^6^ The autopsy findings of this entity are described in only occasional anecdotes.^7-17^ Besides, the histomorphological features at autopsy can be challenging. We hereby describe the complete autopsy findings of a case of AEL in an older man.

## CASE REPORT

A 62-year-old diabetic and hypertensive gentleman, a retired forest surveyor, had insidious onset of breathlessness on exertion, fatigue, and weight loss (~10kg) over the past 3 months. He also complained of intermittent fever, night sweats (x 20 days), and pedal edema (x 7 days). He was referred to our hospital with pancytopenia [Hb 5,4 g/dL (RR: 13,2 to 16,6g/dL), Leukocytes 3.3 x10^9^/L (RR: 4 to 11x10^9^/L), and Platelets 87 x10^9^/L (RR: 135 to 317 x 10^9^/L)]. He had received 4 packed red blood cells (PRBC) and 2 single donors (apheresis) platelets in the past 1 week before admission. There was no documented history of COVID-19, but he received the first COVISHIELD vaccine in October 2021. On examination, patient had an axillary temperature 37,8^0^C, Pulse rate 118/min, respiratory rate 24/min, blood pressure 154/90 mmHg, room air SpO2 98%, ECOG performance status grade 3, pallor and edema. There was no lymphadenopathy. On fundic examination, bilateral severe non-proliferative diabetic retinopathy was noted. There was no hepatomegaly, although a splenic tip was palpable. To summarize, the clinical presentation of severe anemia and B symptoms prompted to the high possibility of hematological malignancy, according to which the patient was investigated further.

An electrocardiogram (ECG) revealed a bi-fascicular block; however, no dynamic changes on serial ECG was noted. On 2D-echocardiography, there was mild left ventricular hypertrophy, mild atrial regurgitation, mild tricuspid regurgitation, mild pulmonary arterial hypertension, grade 1 left ventricular diastolic dysfunction and 62% of left ventricular ejection fraction. All viral serology work-up was negative. The raised serum levels of vitamin B12 and folate were 2000 pg/mL (RR: 200 to 600 pg/mL) and 20 ng/mL (RR: 4.6 to 18.7 ng/mL), respectively. HbA1C was 6.6% after rigorous management. Hospital serum blood glucose level records ranged from 150-300 mg/dL. 24-hour urine protein level was 611 mg per 24 hours. Ultrasonography of the abdomen showed mild fatty infiltration of the liver. Chest X-ray findings suggested pulmonary edema with mild bilateral pleural effusion.

**Figure 1:**
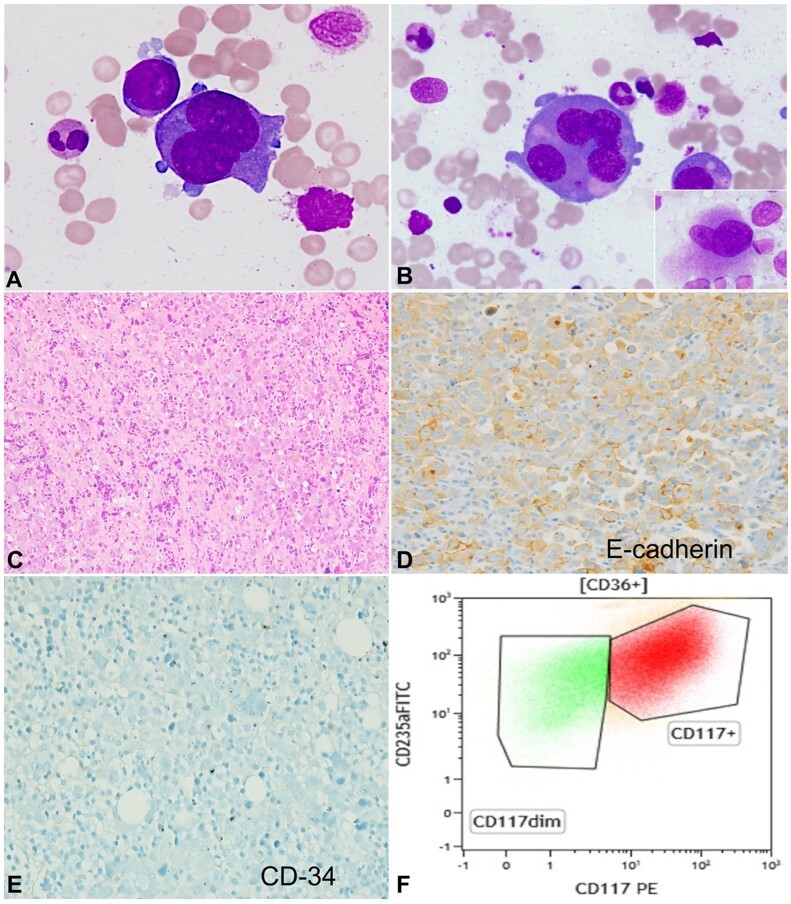
Photomicrograph of the: **A -** bone marrow aspirate showing large and bizarre looking atypical cells with prominent nucleoli and deep basophilic cytoplasm (Giemsa stain; x400); **B -** some multinucleated forms are seen (Giemsa stain; x400). Inset showing dysmegakaryopoiesis; **C** and **D -** the trephine biopsy shows sheets of immature-appearing cells (C; H&E; x200) which are positive for E-cadherin immunostain (D; x400); **E -** CD34 is negative (x400); **F -** flow cytometry plot shows that these atypical cells express CD117 and CD235a.

The peripheral blood smear revealed reduced red blood cell density where the picture was of normocytic normochromic to macrocytic red cells with admixed microcytes and elliptocytes. No significant dysplasia was evident in granulocytes. A bone marrow examination (antemortem) was performed to investigate the cause of pancytopenia. The aspirate was particulate, hypercellular, and showed M:E ratio of 1.2:1. The aspirate showed the presence of large bizarre-looking atypical cells (~16% of all nucleated cells) with coarse chromatin, 2-5 prominent nucleoli, and deeply basophilic cytoplasm (Figure 1A). Occasional multinucleated forms were also noted (Figure 1B). Significant dysmegakaryopoiesis was present. Trephine biopsy was markedly hypercellular (cellularity 90-95%) with large immature cells, focally forming sheets, and scattered dysplastic megakaryocytes (Figure 1C). The morphological differential diagnoses, at this time, included anaplastic myeloma, lymphoma, acute leukemia (especially the rare erythroid and megakaryoblastic variants), histiocytic sarcoma, and metastatic carcinoma. The immunohistochemistry performed on the trephine biopsy showed that the abnormal cells expressed E-cadherin (Figure 1D), but were negative for CD45, CD3, CD20, CD68, CD138, CD34 (Figure 1E), and MPO.

Reticulin stain showed mild fibrosis (1+). Perls’ stain showed 3+ iron deposition. Flow cytometry from the bone marrow aspirate revealed ~4.6% erythroid precursors showing abnormal maturation patterns, with co-expression of early-stage (CD117) and late (CD235a) erythroid markers (Figure 1F).

Fluorescence in situ hybridization showed no evidence of MDS-related abnormalities (-5/del(5q), -7/ del(7q), trisomy 8, and del(20q)). However, three to six copies of multiple genes (*BCR*, *ABL1*, *RBM15*, *MKL1*, *MECOM*) were noted in multiple loci, suggesting near-tetraploidy. Though a definite diagnosis was not made at this point, chiefly due to the small percentage of atypical cells, the differential diagnoses were conveyed to the patient, who was made aware of the guarded prognosis. The patient was lost to follow-up for approximately four weeks following this initial assessment. He returned to the institute with worsened symptoms, and a repeat bone marrow examination was performed. The bone marrow aspirate was aparticulate and hemodiluted with M:E ratio of 1:1.9, and the abnormal cells had now increased to 48%. The trephine biopsy showed sheets of these cells, and the normal hematopoietic elements were markedly reduced. Immunohistochemistry on the repeat biopsy showed that the cells were E-cadherin positive, focal EMA positive, CD61 negative, CK negative, S-100 negative, and chromogranin negative. Targeted resequencing for myeloid mutations revealed *TP53* mutation with high variant allele frequency and *DNMT3A* mutation. The atypical cells fitted best with the profile of early erythroid precursors, and a diagnosis of AEL was made.

He was initially managed along febrile neutropenia with the stepwise escalation of antibiotics (cefoperazone-sulbactam, meropenem, vancomycin, and posaconazole). He required insulin for blood glucose management. He developed hypoxia which was attributed to anemic heart failure and managed with packed red blood cell (PRBC) transfusions, diuretics, and low-flow supplementary oxygen. His general condition deteriorated over the next few days, and he succumbed to the illness on day 7 post-admission (slightly less than a month after he came to medical attention).

## AUTOPSY FINDINGS

A complete autopsy was conducted two hours post-demise after obtaining informed consent from the deceased’s next-to-kin. Each of the pleural cavities yielded 100 ml of straw-colored fluid. Pericardial and peritoneal cavities fluid were within normal limits.

**Figure 2:**
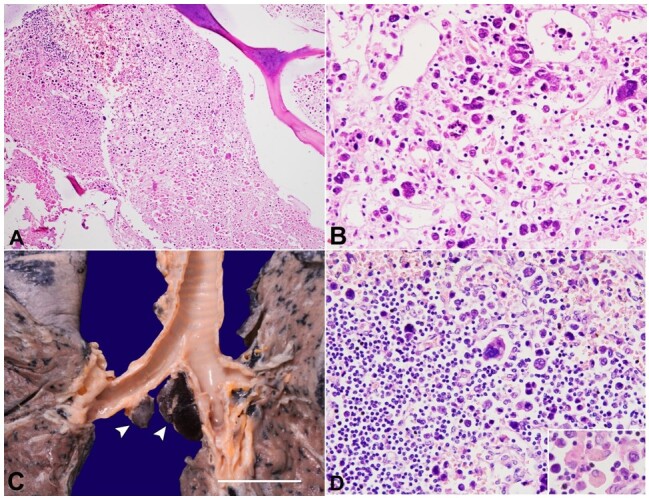
Photomicrograph of: **A -** the postmortem marrow appeared necrotic in 90-95% area with a few viable pockets of viable cells (H&E; x200); **B -** these cells depict marked pleomorphism and contain uni- to multi-lobed nuclei, irregular nuclear membrane, nuclear hyperchromasia, inconspicuous nucleoli, and a moderate amount of cytoplasm. Many cells appeared bizarre and multinucleated (H&E; x400); **C -** on gross, hilar and subcarinal mediastinal lymph nodes (arrowheads) are enlarged and ranges in size from 0.5 to 1.5cm in maximum dimensions (scale bar= 2 cm); **D -** the subcapsular sinuses are distended by the similar atypical cells (H&E; x200). Inset showing hemophagocytosis.

The postmortem bone marrow biopsy showed necrosis involving 90-95% area with a few pockets of viable cells (Figure 2A). These cells were dyscohesive, arranged in diffuse sheets, and showed marked pleomorphism. The individual cells were of variable sizes and shapes, ranging from 2-6 times a mature lymphocyte’s size (Figure 2B). They contained uni- to multi-lobed nuclei, irregular nuclear membrane, nuclear hyperchromasia, inconspicuous nucleoli, and moderate cytoplasm. Many cells appeared bizarre and multinucleated. The mitosis was brisk, often atypical, and numerous apoptotic bodies were noted. Reticulin stain demonstrated grade 2-3 fibrosis in the bone marrow.

The hilar, parahilar, subcarinal, paratracheal, mediastinal, and mesenteric lymph nodes were slightly enlarged, ranging from 0.5-1.5 cm in diameter (Figure 2C). On microscopy, the lymph nodes showed distension of the sinuses by diffuse sheets of similar atypical cells, while the native reactive follicles were preserved (Figure 2D). Extramedullary hematopoiesis with dysplastic megakaryocytes and occasional hemophagocytosis was noted.

**Figure 3:**
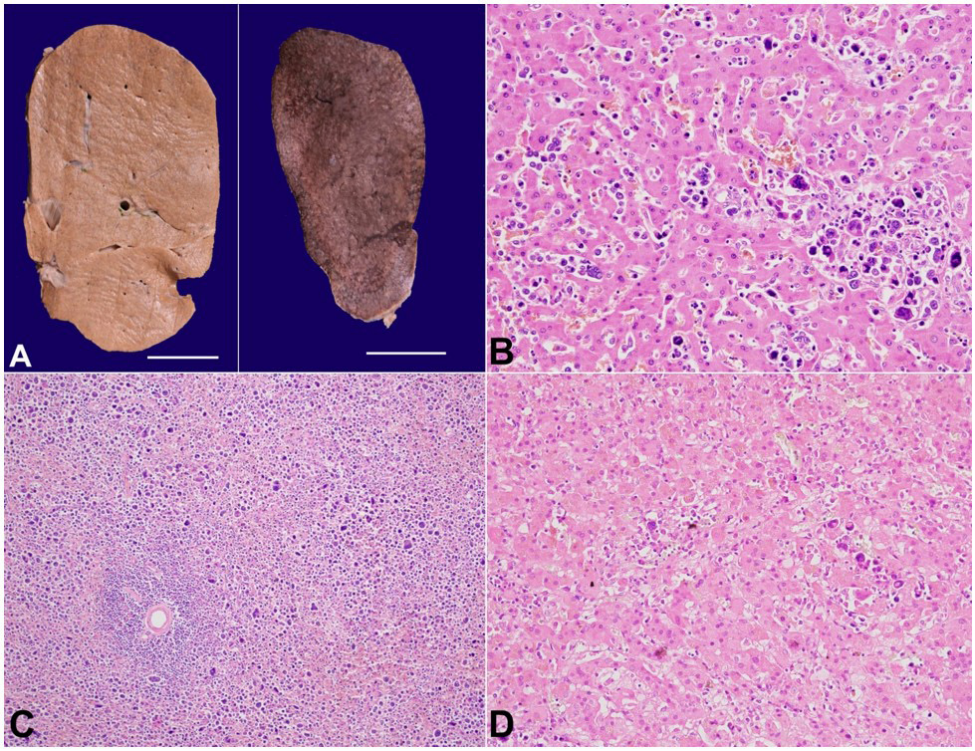
**A -** gross view of the liver (left) (scale bar= 2 cm) and spleen (right) (scale bar= 2 cm). On slicing, the diffusely enlarged liver and spleen show no definite nodularity; **B -** photomicrograph of the liver parenchyma showing predominant intra-sinusoidal pattern of infiltration by the atypical cells (H&E; x200); **C -** the red pulp in spleen shows diffuse expansion due to infiltration (H&E; x100); **D -** the adrenal gland showing evidence of leukostasis (H&E; x200).

The liver was firm, weighed 1600g (mRR: 1450g), and showed diffuse enlargement without nodular formation (Figure 3A). The extrahepatic biliary tree, extrahepatic portal vein, inferior vena cava, and portal vein were unremarkable. Microscopy showed a predominantly intra-sinusoidal pattern of liver infiltration by the atypical cells. These atypical cells infiltrated a few portal tracts with preservation of the ductal and vascular structures (Figure 3B). The spleen weighed 480g (mRR: 114g) and was diffusely enlarged without any grossly visible mass lesion. On microscopy, the spleen showed diffuse expansion of the red pulp by the atypical cells with relative attenuation of the white pulp (Figure 3C). The liver and spleen showed evidence of extramedullary hematopoiesis with dysplastic megakaryocytes. Mesenteric fat also showed infiltration by these atypical cells. The pulmonary capillaries, renal peritubular capillaries, and adrenal sinuses showed focal features of leukostasis (Figure 3D).

**Figure 4:**
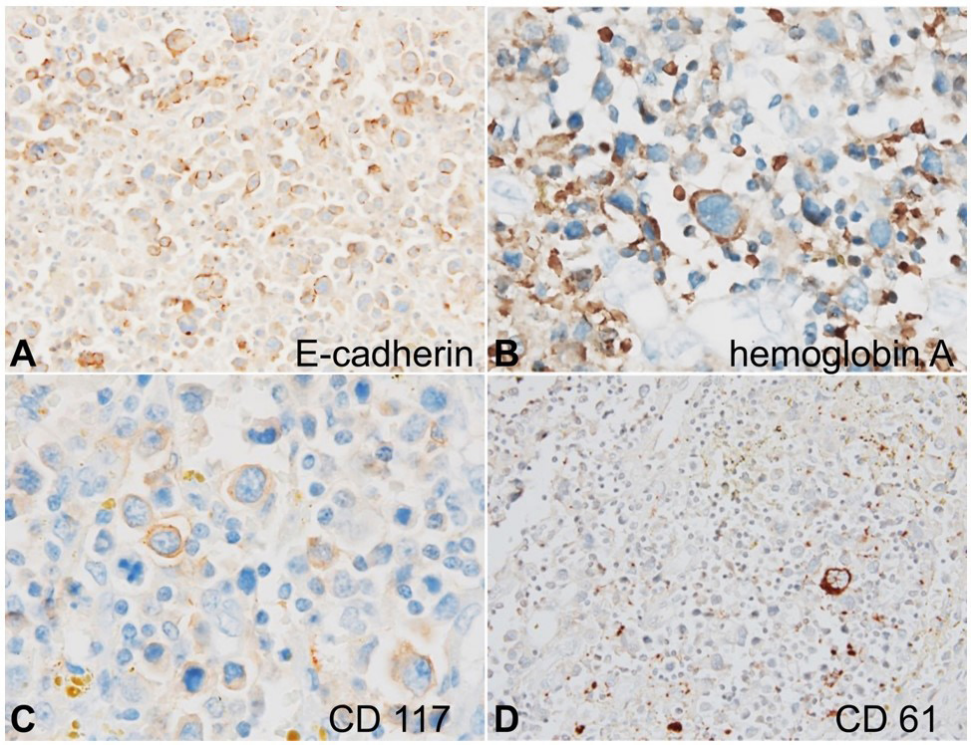
Photomicrograph of the Immunohistochemistry panel of the atypical cells. **A -** E-cadherin shows membranous positivity (x200); **B -** hemoglobin A shows membrane-cytoplasmic positivity (x400); **C -** CD117 shows membrane-cytoplasmic positivity (x400); **D -** CD61 is negative but highlights megakaryocytes (x200).

The atypical cells were negative for PAS stain. They were immunopositive for E-cadherin (diffuse variable intensity membranous) (Figure 4A), hemoglobin A (focal variable intensity membrane-cytoplasmic) (Figure 4B), and CD117 (focal variable intensity membrane-cytoplasmic) (Figure 4C). EMA positivity was very focal. Pan-CK, CD45, CD30, CD20, CD3, CD61 (Figure 4D), MUM1, CD163, CD68, and MPO were negative.

The kidneys weighed 220g (mRR: 313g) and showed gross and microscopic changes of benign nephrosclerosis along with class II glomerular changes (ISN-RPS) of diabetic nephropathy. The abdominal aorta showed complicated atherosclerosis with patent renal artery ostia and patent renal arteries. The heart weighed 350g (mRR: 325g). There was complicated atherosclerosis of the arch of the aorta and descending aorta with concentric left ventricular hypertrophy and triple vessel coronary artery disease (25-50% obliteration). Lungs weighed 650g (mRR: 820g) and showed pleural-based and intra-parenchymal mixed dust pneumoconiosis nodules. There were changes in pulmonary arteriopathy in the form of prominent intra-acinar arterioles, concentric medial hypertrophy, and eccentric intimal fibrosis of the preacinar arterioles. There was evidence of leukostasis involving pulmonary capillaries, renal peritubular capillaries, and adrenal sinusoids. The brain, pancreas, thyroid, skin, testis, gastrointestinal tract, and urinary bladder were normal on gross and microscopy.

Thus, the final autopsy diagnosis in this patient was AEL with myelonecrosis, multiorgan dissemination (liver, spleen, lymph node, and mesenteric fat), and leukostasis. In addition, there was extramedullary hematopoiesis (liver, spleen, lymph node), arterionephrosclerosis, diabetic nephropathy (ISN-RPS class II), left ventricular hypertrophy, mixed dust pneumoconiosis, and pulmonary arteriopathy.

## DISCUSSION

Presently, AEL is characterized as the neoplastic proliferation of immature cells of a pure erythroid lineage that shows maturation arrest with no significant myeloblastic component.^3^ In addition, the expression of erythroid-specific antigens and the presence of biallelic *TP53* alterations along with complex/monosomal karyotypes may contribute to establishing the diagnosis. Diagnosing this entity correctly is of utmost importance because of its rarity, close histomorphological resemblance to other hematopoietic and non-hematopoietic malignancies, rapid clinical course, and non-availability of standardized treatment, and dismal survival outcome. The rapid spread of the disease in multiple viscera with subsequent visceral failure highlights the disease’s natural history and is responsible for the dismal prognosis. The rarity of the disease, the advances in the understanding of the molecular pathology of acute leukemias, and the evolving definition of the entity are the major highlights of this entity. Therefore, the autopsy findings, crucial in understanding the natural history of the disease, are depicted in only a few case reports. A thorough PubMed search of the English literature, revealed about a dozen cases of AEL showing multiorgan dissemination.^7-17^ The rarity of this entity, with only a few case reports depicting the autopsy pathology alongside its challenging histomorphology, are the major points of discussion in the index case.

Recently, the literature has witnessed two types of classification for the hematolymphoid neoplasm: World Health Organization (WHO) and International Consensus Classification (ICC).^3,18^ The PEL is designated as a separate entity in the latest WHO edition; however, it falls under the distinct category of myeloid neoplasms with mutated TP53 per the ICC criteria. In addition to PEL, the latter group also include MDS and MDS/AML. The reason behind this more genetically defined group is to emphasize the more unified treatment strategy as they behave very aggressively irrespective of the blast count. In making PEL diagnosis, the WHO classification rather than ICC utilizes more uniformly the correlation of clinical, unique morphology and immunophenotypic features, flow cytometry findings, and genetic details. Thus, we have followed the latest 5^th^ edition WHO strict definition of AEL, which has evolved significantly over the last few decades. In the Index patient, the first biopsy material revealed the possibility of MDS, which later, based on clinical-pathological-molecular correlation, turned out to be PEL on the second biopsy.

The pathogenesis of AEL is unclear; however, the blockade of erythroid differentiation in murine erythroid progenitors is mediated by aberrant expression of key transcriptional regulators (SKI, ERG, and ETO2) may play a role.^19^ The mean age of presentation is usually 60 years with male preference, similar to the index case. Clinically, the patient suffers from pancytopenia due to extensive bone marrow replacement. Hepatosplenomegaly may also be seen in a subset of cases. The present case also presented with pancytopenia and mild splenomegaly. The bone marrow biopsy appears hypercellular and usually shows near-total replacement (≥80%) by the erythroid cells that mostly contain increased immature precursors (erythroblasts or normoblasts; ≥30%) with a subsequent reduction in the myeloid and the megakaryocyte lineages. Dysmegakaryopoiesis is often a feature of AEL, and the dysplastic megakaryocytes often require confirmation by immunohistochemistry (CD61 immunostain was performed in the index case). CD71 (iron transporter), CD117, E-cadherin, CD235 (Glycophorin A), Hemoglobin, ferritin, and α-hemoglobin stabilizing protein are a few other immunophenotypic markers that aid in the diagnosis of the immature erythroid cells. Nevertheless, these markers are non-specific for erythroid lineage and do not help in distinguishing the reactive and neoplastic erythroid cells.^3^

The reactive and neoplastic conditions showing erythroblast proliferation need to be excluded before making a final diagnosis of AEL. The reactive erythroid hyperplasia can be seen in megaloblastic, hemolytic anemia and secondary to erythroid growth factor administration. Cytogenetic alteration and *TP53* mutation are not seen in the above-mentioned reactive conditions. *TP53* mutation was detected in our case, which ruled out the possibility of reactive erythroid hyperplasia. MDS differs from AEL in sequential maturation, with later-stage erythroblasts as a predominant component. Myeloid neoplasms post-cytotoxic therapy was ruled out as no history of therapy was present. In addition, the index case contained no significant amount of myeloblasts (≥20%), excluding the possibility of acute myeloid leukemia, myelodysplasia-related (AML-MR). The other differentials, namely acute megakaryoblastic leukemia, lymphoblastic leukemia, lymphoma, anaplastic myeloma, histiocytic sarcoma, and metastatic tumors, were ruled out based on the correlation of morphology (erythroid precursors), immunophenotype (CD117+, Hemoglobin A+, and E-cadherin+), and flow cytometry findings (abnormal erythroid maturation). The histopathology suggested features of poorly differentiated malignancy with dyscohesive bizarre cells in diffuse sheets. CD45 negativity and E-cadherin positivity vis-à-vis such morphology may dictate the diagnosis of a poorly differentiated carcinoma, especially in small biopsies. Therefore, a battery of immunopanel must be employed for the correct histopathologic diagnosis of AEL in an unsuspected case.

AEL usually has a complex karyotype with multiple structural abnormalities, often with del(5q) and del(7q).^20^ It shows a high occurrence of *TP53* mutations similar to the index case.^4^ Recently, a de novo *JAK2* R683S mutation was detected in 78-year-old female with PEL.^21^ The AEL cases with multi-organ dissemination, so-called erythroblastic sarcoma, usually shows extramedullary infiltration by the immature blast cells with predominantly hepatic involvement. The other organs include the spleen, abdominal lymph nodes, kidneys, adrenal glands, and, on rare occasions, the periorbital soft tissue.^7-17^ Similarly, the index case depicted infiltration into the liver, spleen, and lymph nodes. The leukostasis was noted in the lungs, kidneys, and adrenal glands. In addition, extramedullary hematopoiesis was also evident in the liver, spleen, and lymph nodes. Due to the non-availability of definite treatment guidelines, the patients may be treated with bone marrow transplants with high-intensity chemotherapy or stem cell transplants. Erythropoietin and granulocyte colony-stimulating factor may be considered in elderly patients.

In conclusion, AEL is an uncommon aggressive hematolymphoid malignancy. The strict definition of AEL has evolved immensely in the last couple of decades. The differential diagnoses are many and include various hematological and non-hematological malignancies and often pose a diagnostic challenge. This case report highlights the autopsy pathology along with the relevant differential diagnoses.
